# TCONS_00230836 silencing restores stearic acid-induced β cell dysfunction through alleviating endoplasmic reticulum stress rather than apoptosis

**DOI:** 10.1186/s12263-021-00685-5

**Published:** 2021-05-22

**Authors:** Rui Guo, Yunjin Zhang, Yue Yu, Shenghan Su, Qingrui Zhao, Xia Chu, Shenglong Li, Huimin Lu✉, Changhao Sun

**Affiliations:** 1 Department of Nutrition and Food Hygiene (National Key Discipline), Public Health College, Harbin Medical University, Harbin, Hei Longjiang province, 150081 People’s Republic of China; 2 General Surgery Department, The Second Affiliated Hospital of Harbin Medical University, Harbin, China

**Keywords:** Stearic acid, Pancreatic β cell, LncRNA, Endoplasmic reticulum stress

## Abstract

**Background:**

Chronic exposure of pancreatic β cells to high levels of stearic acid (C18:0) leads to impaired insulin secretion, which accelerates the progression of type 2 diabetes mellitus (T2DM). Recently, long noncoding RNAs (lncRNAs) were found to participate in saturated fatty acid-induced metabolism dysfunction. However, their contribution to stearic acid-induced β-cell dysfunction remains largely unknown. This study evaluated the possible role of the lncRNA TCONS_00230836 in stearic acid-stimulated lipotoxicity to β cells.

**Method:**

Using high-throughput RNA-sequencing, TCONS_00230836 was screened out as being exclusively differentially expressed in stearic acid-treated mouse β-TC6 cells. Co-expression network was constructed to reveal the potential mRNAs targeted for lncRNA TCONS_00230836. Changes in this lncRNA’s and candidate mRNAs’ levels were further assessed by real-time PCR in stearic acid-treated β-TC6 cells and islets of mice fed a high-stearic-acid diet (HSD). The localization of TCONS_00230836 was detected by fluorescent in situ hybridization. The endogenous lncRNA TCONS_00230836 in β-TC6 cells was abrogated by its Smart Silencer.

**Results:**

TCONS_00230836 was enriched in mouse islets and mainly localized in the cytoplasm. Its expression was significantly increased in stearic acid-treated β-TC6 cells and HSD-fed mouse islets. Knockdown of TCONS_00230836 significantly restored stearic acid-impaired glucose-stimulated insulin secretion through alleviating endoplasmic reticulum stress. However, stearic acid-induced β cell apoptosis was not obviously recovered.

**Conclusion:**

Our findings suggest the involvement of TCONS_00230836 in stearic acid-induced β-cell dysfunction, which provides novel insight into stearic acid-induced lipotoxicity to β cells. Anti-lncRNA TCONS_00230836 might be a new therapeutic strategy for alleviating stearic acid-induced β-cell dysfunction in the progression of T2DM.

**Supplementary Information:**

The online version contains supplementary material available at 10.1186/s12263-021-00685-5.

## Introduction

Chronic exposure to elevated saturated fatty acids (SFAs) of pancreatic β cells is a key trigger of impaired insulin secretion, which is one of the most important characteristics of type 2 diabetes mellitus (T2DM). Evidence accumulated to date has indicated that palmitic acid-mediated insulin resistance and β cell dysfunction are closely related to T2D M[1–3]. However, little attention has been paid to stearic acid-induced worsening of β cell function. In our previous studies and other research, it has been demonstrated that, whether in the postprandial serum of T2DM patients or in the fasting serum of high-fat-diet-fed mice [4–7], only stearic acid levels were profoundly increased. Meanwhile, a long-term high level of stearic acid exhibited a stronger destructive effect on β cells than that of palmitic acid [5, 8]. Although endoplasmic reticulum (ER) stress [5, 9] and apoptosis [8, 10] are accepted as major contributors to stearic acid-induced β cell dysfunction, the molecular mechanisms involved in this remain largely unclear.

Long noncoding RNAs (lncRNAs) [11], generally defined as RNA molecules >200 nucleotides in length without evident protein-coding capacity, are well known to be involved in diverse gene-regulatory mechanisms such as transcription, imprinting, splicing, protein degradation, and epigenetic marks on chromatin [12, 13], and their dysregulation has been implicated in many diseases, especially in several metabolic diseases [14, 15]. Recently, a number of lncRNAs have been reported to participate in SFA-induced metabolic dysfunction in liver [16], adipocytes [17, 18], skeletal muscle cells [19], and pancreatic β cells [20, 21]. However, there are no data regarding the possible contribution of lncRNAs to stearic acid-induced β cell dysfunction.

Using RNA-sequencing and qRT-PCR, we showed for the first time that the lncRNA TCONS_00230836 was differentially expressed exclusively in stearic acid-treated β-TC6 cells, compared with both palmitic acid-treated cellsand control cells. We investigated the effect of the lncRNA TCONS_00230836 on stearic acid-induced β cell dysfunction by loss-of-function approaches in a cellular model of lipotoxicity. Our experimental results suggested that this lncRNA is a detrimental factor to pancreatic β cells in the presence of stearic acid and that its knockdown could mitigate stearic acid-stimulated impairment of insulin secretion, which would provide a potential target for the prevention and treatment of T2DM.

## Material and methods

### Chemicals

Stock solutions of stearic acid and palmitic acid (Sigma, St. Louis, MO, USA) supplemented with BSA (3 mmol/L fatty acids:1.5 mmol/L BSA) were prepared by dissolving them in ethanol and saponifying them with sodium hydroxide, as described previously [22]. After the sodium salt had dried, it was resuspended in saline and then heated at 80 °C until completely dissolved. Next, 20% (w/v) BSA was added and the mixture was stirred at 50 °C for 4 h. The complex was then sterilized and aliquoted.

### Cell culture

The pancreatic β-TC6 cells was obtained from the Shanghai Academy of Chinese Sciences Cell Library and cultured in Dulbecco’s modified Eagle’s medium (DMEM; Gibco/Life Technologies, Carlsbad, CA, USA), supplemented with 15% fetal bovine serum (FBS; Gibco/Life Technologies), 50 μg/mL streptomycin, 50 IU/mL penicillin (Gibco/Life Technologies), and 1.5 g/L NaHCO_3_. Mouse islets were isolated after collagenase P (Cat. 11213873001; Roche Molecular Biochemicals, Germany) digestion of the pancreas by ductal injection and filtered with hardware cloth (600 μm). Next, islets were purified with different concentrations of Ficoll 400 (Cat. 17-0300-10; Pharmacia). Finally, islets were incubated in RPMI 1640 (Gibco) containing 10% FBS, 0.11 g/L sodium pyruvate, and 100 units/mL penicillin-streptomycin. The β-TC6 cells and islets were incubated with 400 μmol/L stearic or palmitic acid for 24 h.

### RNA sequencing and data analysis

The lncRNA and mRNA high-throughput sequencing was performed by Genewiz (Suzhou, China), processing nine samples from pancreatic β-TC6 cells (3 control, 3 stearic acid, and 3 palmitic acid samples). Differential expression analysis was performed using the DESeq Bioconductor package in R by first transforming the raw count data to log_2_ counts per million reads using the “voom” function (*P* < 0.05).

### Construction of gene co-expression network

The Pearson correlation coefficients (PCC) were performed for lncRNA TCONS_00230836-mRNA pairs, of which the PCC ≥0.950, or PCC ≤ −0.950, *P* < 0.05 were selected to construct the gene co-expression network using Cytoscape software. Each gene corresponds to a node in the network.

### Animal experiments

Twenty male C57BL/6J mice (6 weeks old) were purchased from the Beijing Vital River Laboratory Animal Technology Company (Beijing, China). After adaptive feeding for 1 week, these mice were randomly divided into control and high-stearic-acid diet groups (*n* = 10 per group), according to their body weights. The control diet (1025) and high-stearic-acid diet (HSD) (H10060) were obtained from Beijing HFK Bioscience Co., Ltd. (Beijing, China) (Additional file 1). After 20 weeks of feeding the mice, islets and blood samples were collected for biochemical analysis. Fasting (12 h) serum glucose, total cholesterol, triacylglycerol, high-density-lipoprotein cholesterol, and low-density-lipoprotein cholesterol levels were calculated using an automatic analyzer (Hitachi-7100; Hitachi, Tokyo, Japan), kits for which were purchased from Biosino Biotechnology, Co. (Beijing, China). Serum insulin level was measured using a mouse/rat insulin ELISA kit (Linco Research, St. Charles, MO, USA).

### Serum nonesterified fatty acid profile measurement

Fasting serum nonesterified fatty acids were transformed to fatty acid methyl esters, as described in our previous studies [7, 23]. Gas chromatography–mass spectrometry analysis was performed using a TRACE gas chromatograph with a Polaris Q mass spectrometer (Thermo Finnigan, San Jose, CA, USA). Separation was obtained on a J&W DB-WAX capillary column (30-m, 0.25-mm I.D., 0.25-μm film thickness; Agilent J&W Scientific, Folsom, CA, USA). Heptadecanoic acid (C17:0) was used as an internal standard.

### Cell viability measurements

Cell viability was measured by the assessment of lactate dehydrogenase (LDH) release and CCK 8 assay. For measuring LDH, the culture medium was collected and characterized using LDH assay kit (Thermo Fisher Scientific Inc.). For the CCK 8 assay, we used the Cell Counting Kit 8 (CCK-8, C0038; Beyotime Biotechnology, Shanghai, China). The β-TC6 cells were seeded into each well of a 96-well plate and 100 μL of CCK-8 mixed reagents were added to each well. After 1 h of incubation at 37 °C, absorbance was determined at 450 nm using a microplate reader (SpectraMax M2; Molecular Devices, San Jose, CA, USA).

### Apoptosis assay

The β-TC6 cells were collected and stained with fluorescein isothiocyanate (FITC)-annexin V and propidium iodide (PI), in accordance with the manufacturer’s instructions (Lot#AB116F; Absin, Shanghai, China). The apoptosis rate was analyzed by flow cytometry (LSR Fortessa; BD Biosciences, San Jose, CA, USA).

### Fluorescent in situ hybridization (FISH)

The β-TC6 cells were seeded into a 24-well plate with sterile slides overnight. Cells were rinsed using 1× PBS for 5 min and fixed in 4% formaldehyde at room temperature for 10 min. The β-TC6 cells were washed three times with 1× PBS for 5 min each. Next, the cells were permeabilized in 1× PBS containing 0.5% Triton X-100 for 5 min at 4 °C. After washing the cells with 1× PBS, they were blocked in Blocking Solution and Pre-hybridization (1:99) mixed solution at 37 °C for half an hour. Then, in the dark, discarding the mixed solution, the β-TC6 cells were incubated with a hybridized mixture containing lncRNA Probe Mix or U6/18S at 37 °C overnight. On the second day, the β-TC6 cells were washed at 42 °C with different concentrations of SSC solution, which were also protected from the light. After immersing the β-TC6 cells in 1× PBS for 5 min, they were stained with DAPI staining solution at room temperature for 10 min and then washed with 1× PBS. The slides taken from the 24-well plate were observed with a laser confocal microscope. The whole process was protected from light to prevent quenching. The locked nucleic acid-modified oligonucleotide probe targeting lncRNA TCONS_00230836 and the FISH Kit were purchased from Ribobio Co., Ltd. (Guangzhou, China).

### Transfection procedures

β-TC6 cells were transfected with Smart Silencer for TCONS_00230836 and its negative control (Ribobio Co. Ltd.) using C10511-05 riboFect™ CP Transfection Kit (Ribobio Co., Ltd.)for 6 h, in accordance with the manufacturer’s instructions, prior to incubating in normal culture medium for 24h. Then, discarding the supernatant, β-TC6 cells were treated with stearic acid for another 24 h. The sequences of lncRNA Smart Silencer for mouse TCONS_00230836 are displayed in Table 1, including three siRNA and three antisense oligonucleotides.

**Table 1:** The sequences of lncRNA Smart Silencer for mouse TCONS_00230836

Gene	Sequences
siRNA-1 target sequence	CCTTAATCCCAACCCTCAA
siRNA-2 target sequence	TAGACATAGCCACATGAAA
siRNA-3 target sequence	CCCAATAGTTAATGACAGA
Antisense oligonucleotides target sequence-1	GCTGTCAAAAAGGAATCACA
Antisense oligonucleotides target sequence-2	GGAAATGCAGTGTAGTAGAA
Antisense oligonucleotides target sequence-3	ATGGGCCCACCCTCTAAGAT

### Quantitative real-time polymerase chain reaction (qRT-PCR)

Total RNA was extracted from β-TC6 cells and mouse islets using TRIzol reagent (Invitrogen), in accordance with the manufacturer’s protocol. The qRT-PCR was performed with SYBR Green PCR Master Mix (Applied Biosystems, Foster City, CA, USA), with β-actin as an internal control. All primers used in this study were synthesized by Sangon Biotech Co., Ltd. (Shanghai, China), the sequences of which are listed in Table 2.

**Table 2:** Primer sequences used for qRT-PCR

Gene		sequences (5′ to 3′)
TCONS_00230836	Forward	AACCACAGGCTTCGGGATAGTC
	Reverse	CAAGCATAACAGGCGGGAAGTC
ENSMUST00000074898	Forward	ACCTTAAACGACGAGAAGCAATGG
	Reverse	AGCCAGACACGTAGCCCACACG
ENSMUST00000086399	Forward	TGGTCACCGTTGTGATCCC
	Reverse	TGAGGTCCTCCTTGCATG
ENSMUST00000050785	Forward	CTGTCTGTCTGCCACTCCAT
	Reverse	GCTGGCCAAATAAGAAGAGG
ENSMUST00000124100	Forward	ACAAACCGCAGAGAAACAAA
	Reverse	CCTCCCTCTGCCCTAGTATG
ENSMUST00000092822	Forward	GCAGACCCAGTCGTTGTAC
	Reverse	AAGCCTGTGGCACAACATC
ENSMUST00000021620	Forward	GGCTGAAGACAGTTGTGGAA
	Reverse	GGGTAAATCTTGCCCTTTCA
ENSMUST00000073388	Forward	GAGTCACTTGTCCGCAGCTGTC
	Reverse	TCGCTGTCAGTTAAGTCCAGG
ENSMUST00000159720	Forward	CGCTATTGTCTTCTTGATGGAC
	Reverse	CTTCAACAGTTTCCCTGAGTTG
ENSMUST00000131456	Forward	TGATGAGTAGCGTAAAGTACCC
	Reverse	ATATGAGGCATCGTCAGGTAAG
ENSMUST00000185596	Forward	ATCTCTTTGCCTTCCTCAATCA
	Reverse	GTTTTGATCAGCTCATTCACGT
β-actin	Forward	GGTCAGAAGGACTCCTATGTGG
	Reverse	TGTCGTCCCAGTTGGTAACA

### Western blotting

Cells were gently washed three times in 1× PBS and then 50 μL of intermediate RIPA Lysis Buffer (Beyotime) was added per 24-well plate. Protein concentrations were detected using bicinchoninic acid (BCA) protein assay kits (Cat. No. P0010; Beyotime). Protein samples (approximately 80 μg) were separated by sodium dodecyl sulfate polyacrylamide gel electrophoresis and transferred to polyvinylidene fluoride membranes. The primary antibodies used were as follows: Phospho-PERK (3179S, CST, dilution of 1:1000), PERK (3192, CST, dilution of 1:1000), Phospho-eIF2α (9721, CST, dilution of 1:1000), eIF2α (9722, CST, dilution of 1:1000), Caspase-3 (9662, CST, dilution of 1:1000), Parp-1 (9532, CST, dilution of 1:1000), Bcl-2 (4223, CST, dilution of 1:1000), ATF-6 (65880, CST, dilution of 1:1000), IRE1α (3294, CST, dilution of 1:1000), and β-actin (sc-130657, Santa Cruz, dilution of 1:800). The secondary antibody was anti-rabbit (s3738, Promega, dilution of 1:7500) alkaline phosphatase-conjugated antibody. Protein coloration was performed using the Stabilized Substrate for Alkaline Phosphatase (S3841, Promega) and was screened by the FluorChem R system (ProteinSimple, San Jose, CA, USA).

### Glucose-stimulated insulin secretion assay

To assess glucose-stimulated insulin secretion (GSIS), β-TC6 cells were incubated in secretion buffer [NaCl 129, KCl 4.8, MgSO_4_ 1.2, KH_2_PO_4_ 1.2, CaCl_2_ 2.5, NaHCO_3_ 5.0, and HEPES 10 (all mmol/L) supplemented with 1 mg/mL bovine serum albumin, adjusted to pH 7.4] for an additional 60 min with 2.8 or 20 mmol/L glucose [5]. After collecting the supernatant for insulin measurement, the β-TC6 cells were lysed in intermediate RIPA Lysis Buffer (Beyotime) for later evaluation of the total protein content with BCA protein assay reagent kit (Cat. No. P0010, Beyotime) following the manual. Insulin levels in cell culture medium and β-TC6 cells were measured using a mouse/rat insulin ELISA kit (Linco Research) and standardized by every milligram protein per hour in each well.

### Statistics

All statistical data were analyzed with SPSS software, version 21.0 (SPSS Inc., Chicago, IL, USA), and are reported as mean ± SEM. Two-tailed Student’s *t* test was used to analyze differences between two groups, and one-way ANOVA followed by Student–Newman–Keuls test was used to test differences among three or more groups. *P* values < 0.05 were considered statistically significant.

## Results

### Animal model characteristics

We successfully established a mouse model to mimic the increased proportions of stearic acid in vivo, as shown by a significant increase in circulating stearic acid in HSD-fed mice (Additional file 2). The metabolic characteristics of these animals are displayed in Additional file 3.

### Upregulation of lncRNA TCONS_00230836 expression in stearic acid-treated β-TC6 cells and islets of mice fed a high-stearic-acid diet.

**Fig. 1:**
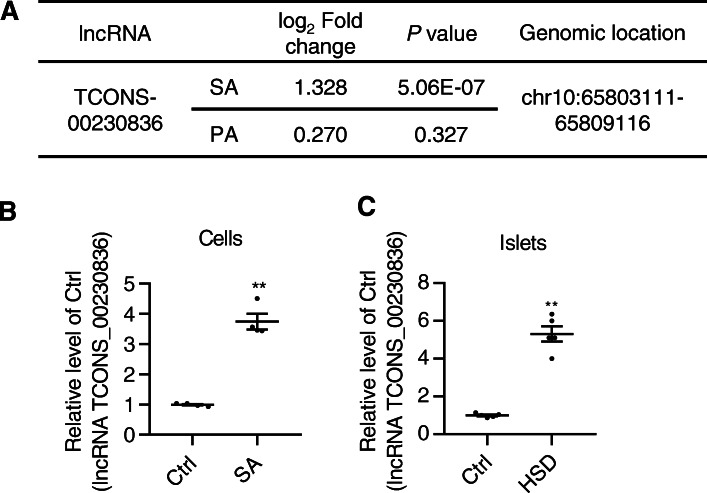
LncRNA TCONS_00230836 upregulation in stearic acid-treated β-TC6 cells and islets of mice fed a high-stearic-acid diet. **a** The studied lncRNA TCONS_00230836 with fold changes, *p* values, and genomic locations in the presence of stearic acid and palmitic acid by RNA sequencing, respectively. (*n*=3 per group) **b** qRT-PCR results verified that the level of the lncRNA TCONS_00230836 was elevated in stearic acid-treated β-TC6 cells. (*n*=4) **c** The expression of the lncRNA TCONS_00230836 was also increased in the islets (n=5) from mice fed a high-stearic-acid diet, as revealed by qRT-PCR. Ctrl control group, SA stearic acid, PA palmitic acid, HSD high-stearic-acid diet. ^**^p < 0.01, versus the Ctrl group

The results obtained by high-throughput RNA-sequencing demonstrated that the lncRNA TCONS_00230836, an intergenic lncRNA located at 65803111 to 65809116 of chromosome 10, was markedly elevated with a 1.328 log_2_ fold change (*P* value: 5.062E-07) in β-TC6 cells exposed to stearic acid and a 0.270 log_2_ fold change (*P* value: 0.327) in the palmitic acid-treated cells, compared with the level in control cells (Fig. 1a). A similar increase in lncRNA TCONS_00230836 level was also revealed in both stearic acid-treated β-TC6 cells and islets of mice fed a high-stearic-acid diet by qRT-PCR. The expression level of the lncRNA TCONS_00230836 was increased by 3.744-fold in stearic acid-treated β-TC6 cells (Fig. 1b) and 5.115-fold in the islets of mice from the high-stearic-acid group (Fig. 1c), compared with that in the corresponding controls.

### Differential expression of lncRNA TCONS_00230836 in mice various tissues

**Fig. 2:**
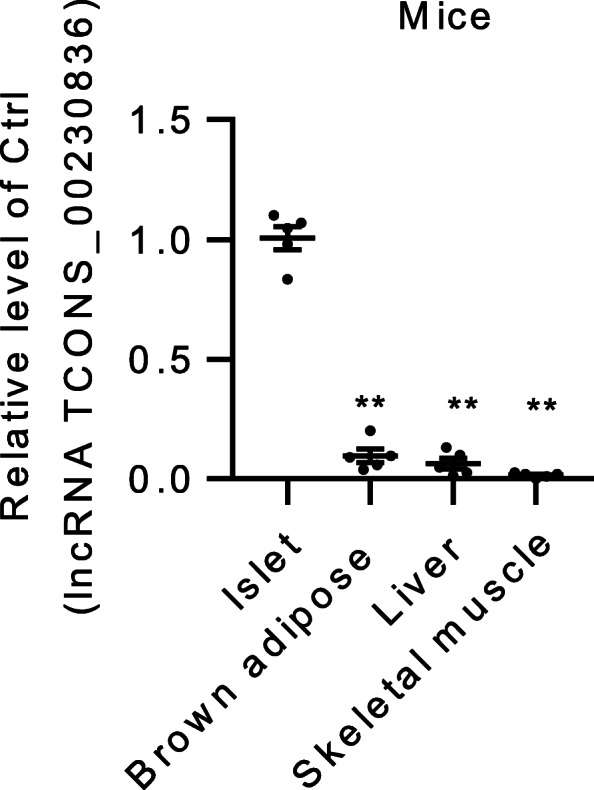
Identification of differential expression of the lncRNA TCONS_00230836 in mice various tissues (islet, brown adipose, liver, and skeletal muscle) fed a chow diet, by qRT-PCR. *n* = 5 mice per group; ^**^p < 0.01 versus the islet group

The selected tissues are closely related to high-fat-diet induced metabolic disorders. Among them, the expression of lncRNA TCONS_00230836 was highest in the islets and lowest in the skeletal muscle of HSD-fed mice (Fig. 2). However, no matching sequence was detected in peritesticular and perirenal adipose tissues (data not shown). In addition to islets, after the treatment with a high concentration of stearic acid, the lncRNA TCONS_00230836 level was also increased in brown adipose, whereas it was decreased in the liver and skeletal muscle of HSD-fed mice (Additional file 4).

### lncRNA TCONS_00230836 is mainly localized in cytoplasm

**Fig. 3:**
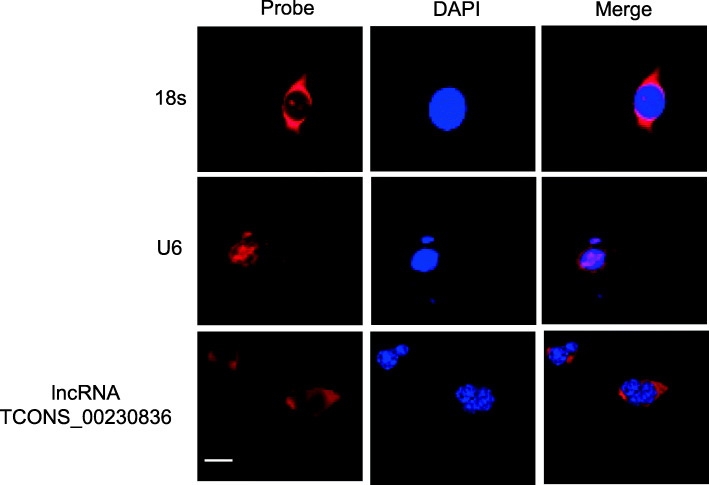
Confocal images of FISH for localization of the lncRNA TCONS_00230836 (red) in β-TC6 cells. Nuclei were stained with DAPI (blue). 18S, probe for 18S rRNA, was used for cytoplasmic localization. U6, probe for U6 snRNA, was taken as a representative of nuclear localization. Scale bar, 10 μm

The confocal images of FISH showed that the lncRNA TCONS_00230836 is mainly distributed in the cytoplasm of β-TC6 cells (Fig. 3).

### Knockdown of lncRNA TCONS_00230836 reversed stearic acid-induced impaired insulin secretion in islets and β-TC6 cells

**Fig. 4:**
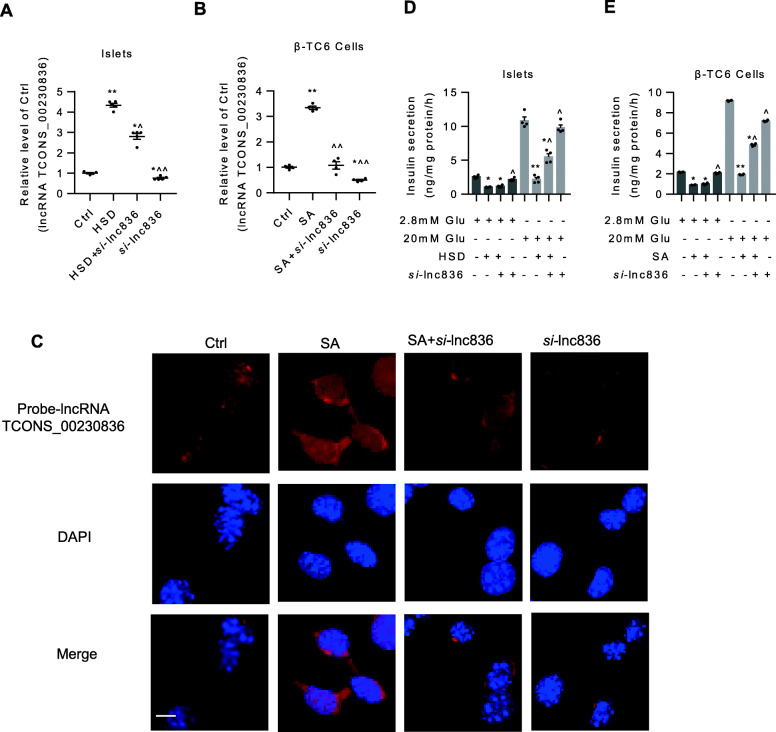
Inhibition of the TCONS_00230836 alleviated stearic acid-induced insulin secretion reduction in islets and β-TC6 cells. **a** The expression of the lncRNA TCONS_00230836 in islets under high-stearic acid diet. **b** The lncRNA TCONS_00230836 level in β-TC6 cells under stearic acid treatment. **c** Representative images of FISH in β-TC6 cells showing the change of lncRNA TCONS_00230836 expression after transfection of the lncRNA TCONS_00230836 Smart Silencer in the presence of stearic acid. Scale bar, 10 μm. **d** Effect of the lncRNA TCONS_00230836 Smart Silencer on GSIS in the islets isolated from mice in the presence of stearic acid. **e** Restoration of stearic acid-induced glucose-stimulated insulin secretion (GSIS) impairment after transfection of the lncRNA TCONS_00230836 Smart Silencer in β-TC6 cells. Ctrl control group, SA stearic acid, *si*-lnc836 Smart Silencer for lncRNA TCONS_00230836. ^*^p < 0.05, ^**^p < 0.01 versus the control group; ^^^*p* < 0.05, ^^^^*p* < 0.01 versus the stearic acid group. *n* =5 and 4 independent animal experiments per group in A and D, respectively. *n* = 4 independent cell cultures per group.

Both in islets and β-TC6 cells (Fig. 4a, b), transfection of the lncRNA TCONS_00230836 Smart Silencer for 24 h efficiently reduced the stearic acid-increased intracellular lncRNA TCONS_00230836 levels. Meanwhile, similar results were consistently observed in β-TC6 cells as indicated by FISH (Fig. 4c). Then, as shown in Fig. 4d, e, stearic acid markedly decreased GSIS in islets and β-TC6 cells, separately. After transfection of the lncRNA TCONS_00230836 Smart Silencer into pancreatic islets and β-TC6 cells, stearic acid-induced GSIS impairment was significantly alleviated. However, no significant alteration was observed in GSIS when the lncRNA TCONS_00230836 Smart Silencer was transfected alone in the absence of stearic acid. Additionally, the insulin content in β-TC6 cells were not significantly changed after transfection of TCONS_00230836 Smart Silencer in the presence or absence of stearic acid. (Additional file 5).

### Inhibition of lncRNA TCONS_00230836 alleviated stearic acid-induced ER stress rather than apoptosis in β-TC6 cells and mice islets

**Fig. 5:**
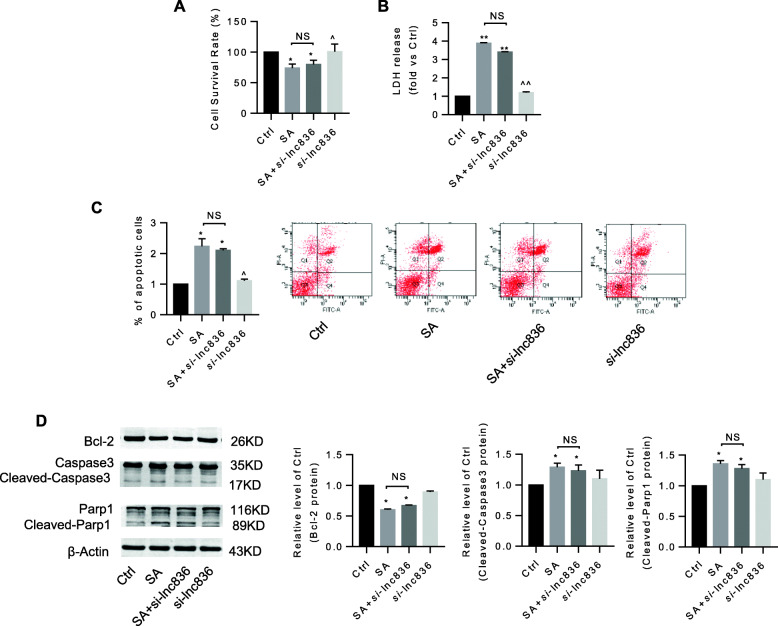
The lncRNA TCONS_00230836 did not significantly reverse stearic acid-induced β-TC6 cell apoptosis. **a**, **b** Effect of the Smart Silencer for TCONS_00230836 on stearic acid-induced cell viability by CCK8 assay and cell death by LDH measurement, separately. **c** FCM revealed that the number of apoptotic cells was not altered after TCONS_00230836 inhibition under stearic acid treatment. **d** No significant changes in the expression of cleaved-caspase3, cleaved-Parp1, and Bcl-2 after inhibition of the lncRNA TCONS_00230836 by western blot analysis. NS not significant, SA stearic acid, *si*-lnc836 Smart Silencer for lncRNA TCONS_00230836. *n* = 3 independent cell cultures per group. ^*^p < 0.05, ^**^p < 0.01 versus the control group; ^^^*p* < 0.05, ^^^^*p* < 0.01 versus the stearic acid group

**Fig. 6:**
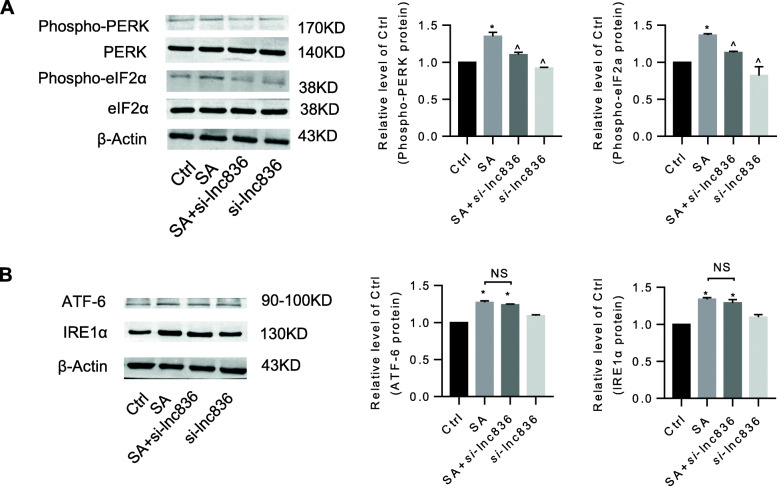
Knockdown of the lncRNA TCONS_00230836 apparently relieved stearic acid-induced ER stress-related proteins in β-TC6 cells. **a** Effect of the lncRNA TCONS_00230836 Smart Silencer on the expression of Phospho-PERK and Phospho-eIF2α proteins in stearic acid-treated β-TC6 cells. **b** Western blot analysis showed the alteration of ATF-6 and IRE1α protein levels after transfection of the TCONS_00230836 Smart Silencer in β-TC6 cells exposed to stearic acid. NS not significant, SA stearic acid, *si*-lnc836 transfection of the Smart Silencer for the lncRNA TCONS_00230836. ^*^p < 0.05 versus the control group; ^^^*p* < 0.05 versus the stearic acid group. *n* = 3 independent cell cultures in each group

**Fig. 7:**
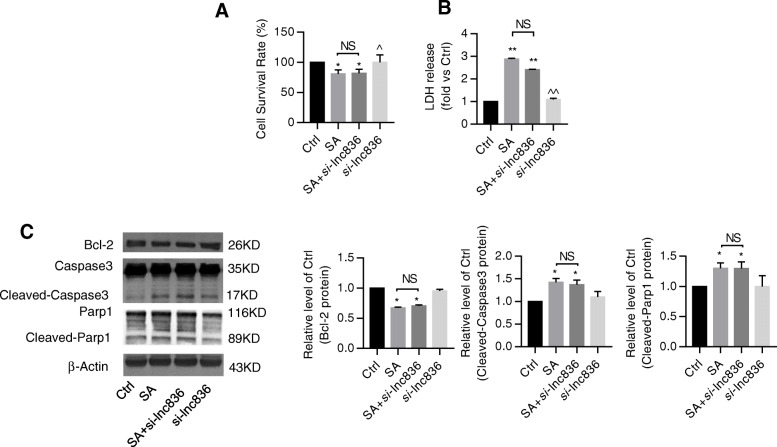
Silencing lncRNA TCONS_00230836 did not markedly alter islet apoptosis exposure to stearic acid. **a**, **b** Assessment of cell viability in HSD mice islets treated with Smart Silencer for TCONS_00230836 by CCK8 assay and cell death by LDH measurement, separately. **c** Western blot analysis verifying the changes in the expression of cleaved-caspase3, cleaved-Parp1, and Bcl-2 after inhibition of the lncRNA TCONS_00230836. NS not significant, SA stearic acid, *si*-lnc836 Smart Silencer for lncRNA TCONS_00230836. *n* = 5 independent animal experiments per group. ^*^p < 0.05, ^**^p < 0.01 versus the control group; ^^^*p* < 0.05, ^^^^*p* < 0.01 versus the SA group

**Fig. 8:**
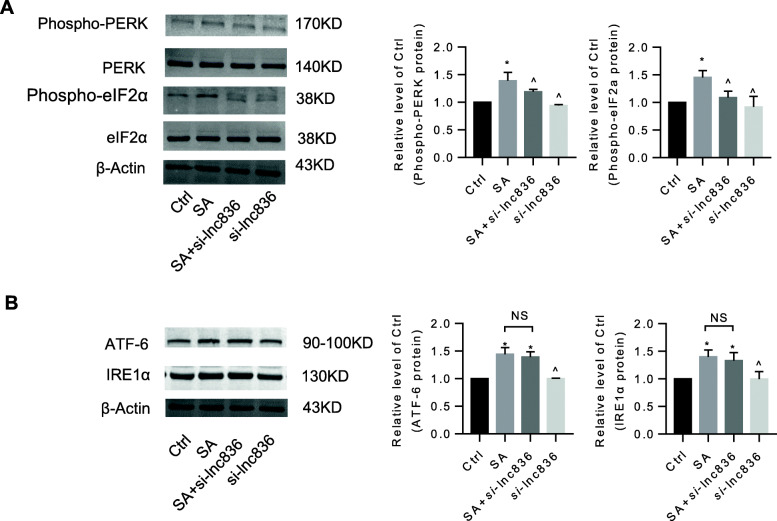
Inhibition of the lncRNA TCONS_00230836 apparently recovered ER stress-related proteins in stearic acid-treated mice islets. **a** The expression of Phospho-PERK and Phospho-eIF2α proteins in mice islet under high-stearic-acid treatment by the lncRNA TCONS_00230836 Smart Silencer. **b** The alteration of ATF-6 and IRE1α protein levels after transfection of the TCONS_00230836 Smart Silencer in islet exposed to high level of stearic-acid. NS not significant, SA stearic-acid, *si*-lnc836 transfection of the Smart Silencer for the lncRNA TCONS_00230836. ^*^p < 0.05 versus the control group; ^^^*p* < 0.05 versus SA group. *n* = 5 independent animal experiments per group

Stearic acid apparently suppressed the cell survival rate and induced cell death in β-TC6 cells. Silencing the lncRNA TCONS_00230836 did not significantly block the stearic acid-stimulated cell viability reduction (Fig. 5a) and cell death (Fig. 5b). Meanwhile, the annexin V-FITC apoptosis assay results showed that lncRNA TCONS_00230836 inhibition did not reduce the stearic acid-increased percentage of apoptotic cells (Fig. 5c). Besides, the elevated protein levels of cleaved-Parp1 and cleaved-Caspase3, and the decreased levels of B-cell CLL/lymphoma 2 in the stearic acid group were not rescued by the lncRNA TCONS_00230836 Smart Silencer (Fig. 5d). However, the expression of the ER stress-related proteins Phospho-PERK and Phospho-eIF2α in stearic acid-treated β-TC6 cells was significantly decreased after inhibition of the lncRNA TCONS_00230836 (Fig. 6a), whereas stearic acid-increased protein levels of ATF-6 and IRE1α were not suppressed after knockdown of TCONS_00230836 (Fig. 6b). Moreover, transfection of the lncRNA TCONS_00230836 Smart Silencer alone did not alter apoptosis and ER stress in β-TC6 cells. In addition, similar results were consistently observed in mice islets (Fig 7a–c, Fig. 8a, b).

### Effect of lncRNA TCONS_00230836 on the expressions of candidate ER stress-related mRNAs

**Fig. 9:**
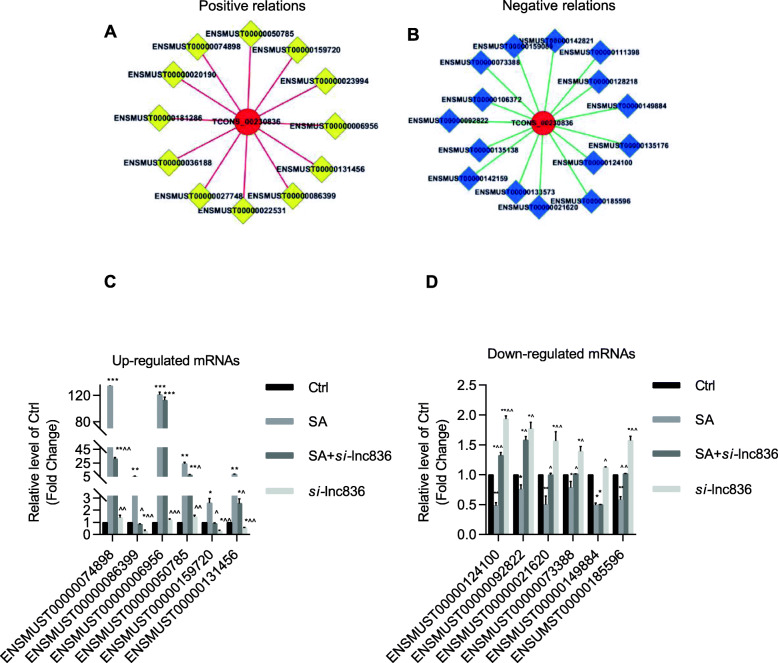
Alterations of candidate ER stress-related mRNAs expressions after transfection of lncRNA TCONS_00230836 Smart Silencer. **a**, **b** The positive and negative co-expression network of lncRNA TCONS_00230836 and target mRNAs (positively related: *Hp*, *Icam1*, *SAA3*, *Lcn2*, *Alpk1*, *Serping1*, *Vnn3*, *Rgs16*, *Gm16685*, *Lats2*, *Zc3h12a*. Negatively related: *Prn*, *Bcas3*, *Otub2*, *Afmid*, *Snapin*, *Srgap2*, *2010107G23Rik*, *Sult1a1*, *Ipo9*, *Gm15350*, *Gm16272*, *Ncor2*) specifically in stearic acid-treated β-TC6 cells. Red circle nodes represent upregulated lncRNA TCONS_00230836. Genes labeled with yellow and blue squares refer to upregulated and downregulated mRNAs, respectively. Red lines represent a positive correlation and green lines refer to negative correlation. **c** Effect of lncRNA TCONS_00230836 Smart Silencer on stearic acid-increased ER stress-related mRNAs expressions. **d** Changes in the downregulated ER stress-related mRNAs after silencing lncRNA TCONS_00230836. n=3,  ^*^p < 0.05, ^**^p < 0.01, ^***^p < 0.001, versus the ctrl group; ^^^*p* < 0.05, ^^^^*p* < 0.01, ^^^^^*p* < 0.001 versus SA group

In order to reveal the potential mRNAs targeted for lncRNA TCONS_00230836 in stearic acid-treated β cell ER stress, the lncRNA TCONS_00230836-mRNAs co-expression network was constructed. Twelve upregulated mRNAs are positively associated with lncRNA TCONS_00230836 (Fig. 9a), and 15 downregulated mRNAs are negatively related to this lncRNA (Fig. 9b). The log_2_ fold change of these upregulated and downregulated differentially expressed mRNAs were listed in Additional file 6. Among these mRNAs, six stearic acid-increased mRNA levels (positively related) (ENSMUST00000074898 [*Hp*], ENSMUST00000159720 [*Alpk1*], ENSMUST00000006956 [*SAA3*], ENSMUST00000086399 [*Icam1*], ENSMUST00000131456 [*Serping1*], and ENSMUST00000050785 [*Lcn2*]) were effectively reversed by lncRNA TCONS_00230836 Smart Silencer, except ENSMUST00000006956 [*SAA3*]. Moreover, after transfection of lncRNA TCONS_00230836 Smart Silencer alone without stearic acid treatment, only *Alpk1*, *Icam1*, and *Serping1* were significantly reduced (Fig. 9c). As for negatively related mRNAs, the expressions of six stearic acid-decreased mRNAs (ENSMUST00000124100 [*Prn*], ENSMUST00000092822 [*Bcas3*], ENSMUST00000021620 [*Otub2*], ENSMUST00000073388 [*Afmid*], ENSMUST00000149884 [*Snapin*], and ENSMUST00000185596 [*Srgap2*]) were markedly rescued after knocking down lncRNA TCONS_00230836, exept for snapin. Meanwhile, these six mRNAs were significantly upregulated after silencing lncRNA TCONS_00230836 in the absence of stearic acid (Fig. 9d).

## Discussion

The prevalence of T2DM has been increasing worldwide, with an estimated 336 million people currently affected [24]. It is well established that exposing pancreatic β cells to an elevated level of stearic acid causes their dysfunction, which is far advanced by the time diabetes is diagnosed clinically [4–6, 8]. Therefore, the optimal management and prevention of T2DM should aim to ameliorate β cell dysfunction at an early stage. However, the effective preventive targets are limited and still need to be comprehensively explored. The present study elucidated the pathophysiological role of lncRNA in stearic acid-treated β cells. Our findings demonstrated that the lncRNA TCONS_00230836 was significantly increased in both stearic acid-induced β-TC6 cells and the islets of mice fed a high-stearic-acid diet. Inhibition of the lncRNA TCONS_00230836 effectively protected against stearic acid-induced β cell dysfunction. Interestingly, after knocking down the lncRNA TCONS_00230836, we found that stearic acid-stimulated β cell ER stress was apparently ameliorated via a PERK/eIF2α-dependent pathway, but no obvious improvement of stearic acid-induced apoptosis was observed.

Although a number of studies have preliminarily explored the mechanisms underlying stearic acid-induced pancreatic β cell damage, the exact molecular targets and associated pathways still need to be established. Recently, accumulating evidence has suggested that noncoding RNAs play an important role in the β cell injury caused by the long-term consumption of a high-fat diet, such as microRNAs [5, 8], circRNAs [25, 26], and lncRNAs [20, 27, 28]. Nevertheless, few studies have focused on the role of lncRNAs in β cell dysfunction upon exposure to elevated levels of SFAs, especially stearic acid, on which no related research has previously been published. In our study, we used high-throughput sequencing and bioinformatic technology to select five lncRNAs that were differentially expressed specifically in stearic acid-treated β-TC6 cells, compared with their levels in both control and palmitic acid groups (data not shown). Among the known lncRNAs, the lncRNA TCONS_00230836 displayed the largest fold increase, with an increase of 1.328 log_2_ fold change in RNA sequencing results, 3.744-fold in stearic acid-induced β-TC6 cells, and 5.115-fold in islets of mice fed a high-stearic-acid diet, as revealed by qRT-PCR.

It has been well clarified that lncRNAs have low sequence conservation and high tissue specificity [29]. Meanwhile, the expression of lncRNAs typically varies more between tissues than for other noncoding RNAs [30–32]. To investigate the difference of sequence conservation and differential expression of the lncRNA TCONS_00230836, we tested it in different tissues (islet, liver, skeletal muscle, brown adipose, peritesticular, and perirenal adipose tissues) which are closely related to T2DM. Our data indicated that there was a highly similar sequence of lncRNA TCONS_00230836 (similarity > 98%) in the liver, brown adipose, and skeletal muscle, but not in peritesticular and perirenal adipose tissues, compared with that in islets. Moreover, the level of this lncRNA was highest in the islets and lowest in the skeletal muscle of high-stearic-acid-fed mice, which suggested that this lncRNA may be specific to and enriched in the islets. This is the first key finding of our study.

To demonstrate the role of the lncRNA TCONS_00230836 in stearic acid-mediated lipotoxicity in β cells, endogenous TCONS_00230836 in β-TC6 cells was abrogated by the application of its Smart Silencer, a highly effective inhibitor that specifically targets lncRNA TCONS_00230836 expressed in both the cytoplasm and the nucleus. We found that stearic acid-impaired GSIS was largely restored after silencing TCONS_00230836, which implied that a TCONS_00230836-mediated mechanism operated in the stearic acid-induced lipotoxicity of β cells. However, how does the lncRNA TCONS_00230836 exert its influence and which process was improved in stearic acid-induced pancreatic β cell injury?

ER stress and apoptosis are very important pathophysiological perturbations and are closely associated with β cell lipotoxicity in the early stage of T2DM; they are not only independent of each other, but also have a close cause-effect relationship [33–37]. To reveal which process was affected by the lncRNA TCONS_00230836, we detected the alterations of ER stress and apoptosis induced by stearic acid after transfection of the Smart Silencer for the lncRNA TCONS_00230836. Our data indicated that transfection of the lncRNA TCONS_00230836 effectively ameliorated stearic acid-stimulated ER stress via a PERK/eIF2α-dependent pathway. As we know, ER stress can transduce apoptotic signals that may eventually result in apoptosis. However, unexpectedly, stearic acid-induced β cell apoptosis was not significantly recovered after knocking down lncRNA TCONS_00230836 expression. Therefore, we speculate that there may be some other key regulatory factors involved in lncRNA TCONS_00230836-mediated β cell ER stress. This is another important finding of our study.

In an effort to reveal the potential target for lncRNA TCONS_00230836 in stearic acid-induced ER stress, we analyzed the mRNAs profile by high-throughput screens in our previous study [38] and constructed the co-expression network between lncRNA TCONS_00230836 and targeted mRNAs which are closely related to ER stress. Then, the top six upregulated mRNAs and another top six downregulated mRNAs were selected for further identification. In the positive-relation group, qPCR results for the mRNA expressions indicated that *Alpk1*, *Icam1*, and *Serping1* are probably involved in the process of stearic acid-induced β cell ER stress associated with lncRNA TCONS_00230836. While in negative-relation group, *Prn*, *Bcas3*, *Otub2*, *Afmid*, and *Srgap2* might play a critical role in stearic acid β cell ER stress closely related to lncRNA TCONS_00230836. Among them, *Afmid* has been reported that they could lead to impaired glucose tolerance in type 2 diabetes [39]. Meanwhile, *Otub2* has been shown to act through the inhibition of NF-κB signaling in type 1 diabetes [40, 41]. Further experiments should be performed to explore the precise regulatory relationship between these candidate mRNAs and lncRNA TCONS_00230836 as well as PERK/eIF2α-dependent signaling in stearic acid-treated β cells.

Our understanding of lncRNAs in stearic acid-induced lipotoxicity to β cells is still in its infancy and several questions remain unanswered. First, we did not assess the role of the lncRNA TCONS_00230836 in stearic acid-induced impairment of insulin secretion in an in vivo experiment. Second, we all know that lncRNAs exert their function through diverse regulatory mechanisms, for example, modulating translation, promoting mRNA degradation, or acting as miRNA sponges. In our previous study [5], we found that stearic acid-induced β cell apoptosis is attributed to miR-34a-5p, which is positively regulated by PERK/p53. While the present study indicated that inhibition of lncRNA TCONS_00230836 siginificantly reversed stearic acid-increased phosphor-PERK level. Therefore, further experiments still need to be performed to analyze the relationship and direct binding site between this lncRNA and miR-34a-5p, as well as other potential TCONS_00230836-related targets in stearic acid-induced β cell dysfunction, which would improve our understanding of the regulatory mechanisms of the lncRNA TCONS_00230836 in stearic acid-induced lipotoxicity to pancreatic β cells.

### Values

We provide evidence that stearic acid can stimulate upregulation of the lncRNA TCONS_00230836 in stearic acid-treated pancreatic β cells. It appears that this lncRNA is specific to and enriched in pancreatic β cells. Suppression of this lncRNA could effectively restore stearic acid-induced impairment of insulin secretion through the amelioration of ER stress rather than apoptosis. Among the three major ER-stress-related sensors, only PERK/eIF2α contributes to this process after knockdown of lncRNA TCONS_00230836. These findings provide novel insight into stearic acid-induced lipotoxicity and the progression of T2DM, whereby lowering the level of the lncRNA TCONS_00230836 might be an effective strategy for improving lipotoxicity in β cells.

## Supplementary Information

**Additional file 1.** The composition of the diet for mice

**Additional file 2.** The composition of fasting serum NEFAs profile in normal and HSD mice at 20

**Additional file 3.** Body weight and serum analysis in normal and HSD mice at 20 weeks.

**Additional file 4.** Alteration of the lncRNA TCONS_00230836 level in brown adipose, liver, and skeletal muscle of mice fed a high-stearic-acid diet. *n* = 5 mice per group. Ctrl, control group; HSD, high-stearic-acid diet. ^*^P < 0.05, ^**^P < 0.01 versus the Ctrl group.

**Additional file 5.** The intracellular insulin content in β-TC6 cells after transfection of the TCONS_00230836 Smart Silencer in the absence or presence of stearic acid. *n* = 3 cell cultures per group. Ctrl, control group; SA, stearic acid, si-lnc836, Smart Silencer for TCONS_00230836.

**Additional file 6.** The log_2_ fold change of up- and down-regulated differentially expressed mRNAs related to lncRNA TCONS_00230836 (PCC ≥0.950, or PCC ≤ -0.950, *P* < 0.05) in stearic acid-induced β-TC6 cells by high-throughput RNA-sequencing.

## Data Availability

All of the data are available with reasonable request from the corresponding authors.

## Abbreviations

CCK8: Cell Counting Kit 8
GSIS: Glucose-stimulated insulin secretion
HSD: High stearic acid diet
LDH: Lactate dehydrogenase
PA: Palmitic acid
qRT-PCR: Quantitative real-time PCR
SA: Stearic acid
SFAs: Saturated fatty acids
T2DM: Type 2 diabetes mellitus
