# Correction to: TCONS_00230836 silencing restores stearic acid-induced β cell dysfunction through alleviating endoplasmic reticulum stress rather than apoptosis

**DOI:** 10.1186/s12263-021-00690-8

**Published:** 2021-07-12

**Authors:** Rui Guo, Yunjin Zhang, Yue Yu, Shenghan Su, Qingrui Zhao, Xia Chu, Shenglong Li, Huimin Lu, Changhao Sun

**Affiliations:** 1 Department of Nutrition and Food Hygiene (National Key Discipline), Public Health College, Harbin Medical University, Harbin, Hei Longjiang province 150081 People’s Republic of China; 2 General Surgery Department, The Second Affiliated Hospital of Harbin Medical University, Harbin, China

**Correction to: Genes Nutr 16, 8 (2021)**

**https://doi.org/10.1186/s12263-021-00685-5**

Following publication of the original article [1], the authors flagged that an earlier version of the chr 7 had been mistakenly written as chr 10 in Fig. 1A and in the “Upregulation of lncRNA TCONS_00230836 expression in stearic acid-treated β-TC6 cells and islets of mice fed a high-stearic-acid diet” section of the Results.

The original article has been updated and the corrected version of Fig. 1 is provided in this correction.

The authors apologize for any inconvenience caused.

**Fig. 1:**
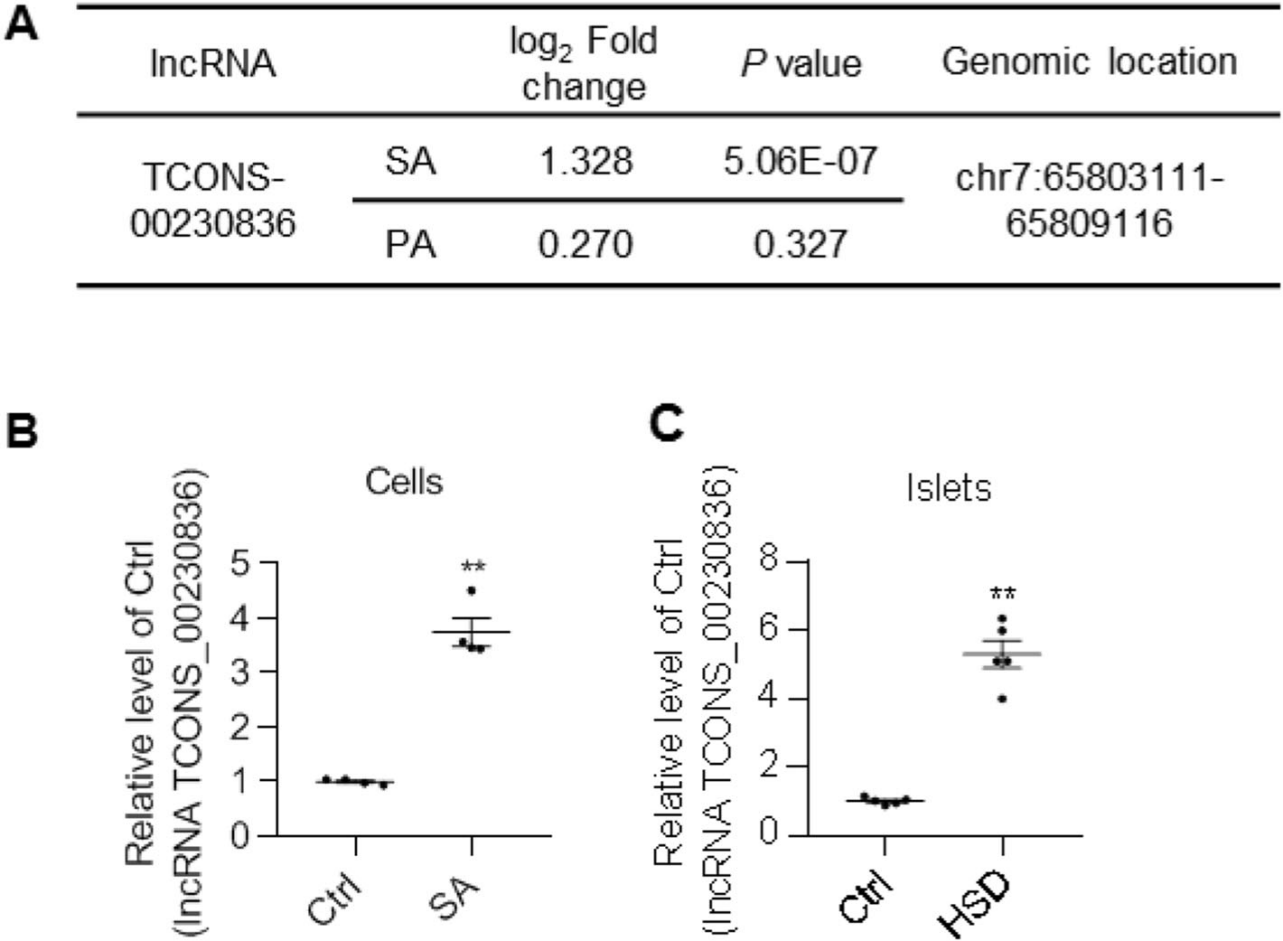
LncRNA TCONS_00230836 upregulation in stearic acid-treated β-TC6 cells and islets of mice fed a high-stearic-acid diet. **a** The studied lncRNA TCONS_00230836 with fold changes, *p* values, and genomic locations in the presence of stearic acid and palmitic acid by RNA sequencing, respectively. (*n*=3 per group) **b** qRT-PCR results verified that the level of the lncRNA TCONS_00230836 was elevated in stearic acid-treated β-TC6 cells. (*n*=4) **c** The expression of the lncRNA TCONS_00230836 was also increased in the islets (n=5) from mice fed a high-stearic-acid diet, as revealed by qRT-PCR. Ctrl control group, SA stearic acid, PA palmitic acid, HSD high-stearic-acid diet. ^**^p < 0.01, versus the Ctrl group
