# Supplementary material for: TCONS_00230836 silencing restores stearic acid-induced β cell dysfunction through alleviating endoplasmic reticulum stress rather than apoptosis
Source: Genes Nutr. 2021 May 22;16:8. doi: 10.1186/s12263-021-00685-5 (PMC8140511; doi:10.1186/s12263-021-00685-5)
Supplement: Supplementary file 1 — Additional file 1. The composition of the diet for mice [file 12263_2021_685_MOESM1_ESM.docx]

**Additional file 1**

The composition of the diet for mice

| Ingredients | Low fat diet | High SA diet |
| --- | --- | --- |
| Cornstarch (g/kg) | 367.5 | 40 |
| Casein (g/kg) | 213.465 | 258.45 |
| Dextrinized cornstarch (g/kg) | 121.53 | 121.53 |
| Sucrose (g/kg) | 88.91 | 88.91 |
| Lard (g/kg) | 0 | 316.6 |
| Soybean oil (g/kg) | 51 | 32.31 |
| Fiber (g/kg) | 40 | 64.61 |
| Mineral mix (AIN-93G-MX) (g/kg) | 12.92 | 12.92 |
| Vitamin mix (AIN-93G-VX) (g/kg) | 12.92 | 12.92 |
| L-cystine (g/kg) | 3.88 | 3.88 |
| Choline bitartrate (g/kg) | 2.58 | 2.58 |
| Calcium carbonate (g/kg) | 16.8 | 16.8 |
| Calcium hydrogen phosphate (g/kg) | 7.11 | 7.11 |
| Potassium citrate(g/kg) | 21.32 | 21.32 |
| Edible blue dye (g/kg) | 0.065 | 0.065 |
| Energy (kcal/kg) | 3851.82 | 5242.95 |

SA: stearic acid
