# Supplementary material for: TCONS_00230836 silencing restores stearic acid-induced β cell dysfunction through alleviating endoplasmic reticulum stress rather than apoptosis
Source: Genes Nutr. 2021 May 22;16:8. doi: 10.1186/s12263-021-00685-5 (PMC8140511; doi:10.1186/s12263-021-00685-5)
Supplement: Supplementary file 2 — Additional file 2. The composition of fasting serum NEFAs profile in normal and HSD mice at 20 [file 12263_2021_685_MOESM2_ESM.docx]

**Additional file 2**

The composition of fasting serum NEFAs profile in normal and HSD mice at 20 weeks

| FFAs (μg/ml) | Normal mice | HSD mice |
| --- | --- | --- |
| C14:0, MA  (Myristic acid) | 4.143±0.524 | 7.334±0.414^***^ |
| C16:0, PA  (Palmitic acid) | 145.595±8.251 | 296.947±10.561^***^ |
| C16: 1, PLA  (Palmitoleic acid) | 8.024±1.529 | 3.865±0.198^**^ |
| C18: 0, SA  (Stearic acid) | 62.899±8.111 | 155.962±15.305^***^ |
| C18: 1, OA  (Oleic acid) | 67.062±9.186 | 116.553±4.967^***^ |
| C18: 2, LA  (Linoleic acid) | 119.405±11.308 | 141.783±12.717^*^ |
| γ-C18:3, γ-LNA  (γ-Linolenic acid) | 5.895±0.667 | 8.038±0.763^**^ |
| C18: 3, LNA  (Linolenic acid) | 86.022±13.233 | 36.206±14.455^**^ |
| C20: 2, EDA  (Eicosadienoic acid) | 7.951±0.101 | 8.040±0.177 |
| C20: 4, AA  (Arachidonic acid) | 83.352±4.659 | 102.610±2.792^***^ |
| C20: 5, EPA  (Eicosapentaenoic acid) | 7.753±1.185 | 2.476±0.774^***^ |
| C22: 5, DPA  (Docosapentaenoic acid) | 1.817±0.205 | 1.859±0.478 |
| C22: 6, DHA  (Docosahexaenoic acid) | 156.332±13.393 | 155.588±6.356 |
| Saturated fatty acids | 208.495±12.661 | 452.909±20.179^***^ |
| Total fatty acids | 756.251±33.237 | 1037.261±48.279^***^ |
| Percentage of SA (%) | 8.324±1.069 | 15.013±0.896^***^ |
| Percentage of PA (%) | 19.297±1.607 | 28.663±1.347^***^ |
| PA/SA ratio | 2.343:1 | 1.917:1^*^ |

Values are means ± SEM. *n* = 10 mice in each group. ^*^*p* < 0.05, ^**^*p* < 0.01, ^***^*p* < 0.001, compared with the value of normal mice.
