# Supplementary material for: TCONS_00230836 silencing restores stearic acid-induced β cell dysfunction through alleviating endoplasmic reticulum stress rather than apoptosis
Source: Genes Nutr. 2021 May 22;16:8. doi: 10.1186/s12263-021-00685-5 (PMC8140511; doi:10.1186/s12263-021-00685-5)
Supplement: Supplementary file 3 — Additional file 3. Body weight and serum analysis in normal and HSD mice at 20 weeks. [file 12263_2021_685_MOESM3_ESM.docx]

**Additional file 3**

Body weight and serum analysis in normal and HSD mice at 20 weeks.

| Characteristics | Normal mice | HSD mice |
| --- | --- | --- |
| Body weight (g) | 32.283±0.831 | 48.153±1.003^***^ |
| Glucose (mmol/l) | 4.554±0.416 | 11.555±1.681^***^ |
| Insulin (ng/ml) | 69.3941±6.271 | 117.402±9.166^***^ |
| TC (mmol/l) | 2.614±0.347 | 6.209±0.501^***^ |
| TG (mmol/l) | 0.840±0.026 | 1.344±0.099^**^ |
| HDL-C (mmol/l) | 3.331±0.391 | 3.171±0.196 |
| LDL-C (mmol/l) | 0.242±0.030 | 1.304±0.175^***^ |
| Food intake (g) | 4.177±0.939 | 3.941±0.319 |

After feeding for 20 weeks, the food intake, body weight, fasting blood glucose, insulin, TC, TG, LDL-C and HDL-C levels were measured in C57BL/6J mice. All the parameters were detected and calculated in the fasting state. Display mode was mean ± SEM, n=10 mice per group. ^**^*p* < 0.01, ^***^*p* < 0.001, compared with the value of normal mice.
