# Supplementary material for: TCONS_00230836 silencing restores stearic acid-induced β cell dysfunction through alleviating endoplasmic reticulum stress rather than apoptosis
Source: Genes Nutr. 2021 May 22;16:8. doi: 10.1186/s12263-021-00685-5 (PMC8140511; doi:10.1186/s12263-021-00685-5)
Supplement: Supplementary file 4 — Additional file 4. Alteration of the lncRNA TCONS_00230836 level in brown adipose, liver, and skeletal muscle of mice fed a high-stearic-acid diet. n = 5 mice per group. Ctrl, control group; HSD, high-stearic-acid diet. *P < 0.05, **P < 0.01 versus the Ctrl group. [file 12263_2021_685_MOESM4_ESM.docx]

**Additional file 4**


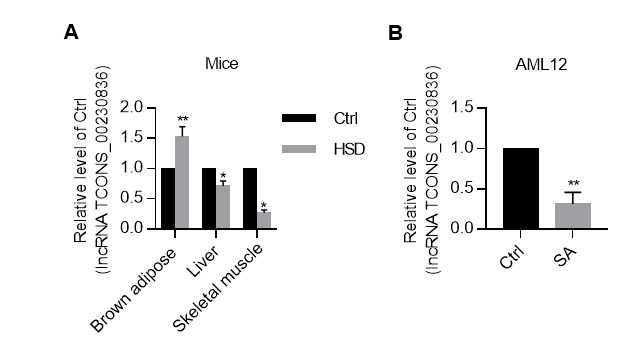


Changes in the expression of lncRNA TCONS_00230836 in various mice tissues fed a high-stearic-acid diet and AML12 cells exposed to stearic acid.

(A) Alteration of the lncRNA TCONS_00230836 level in brown adipose, liver, and skeletal muscle of mice fed a high-stearic-acid diet. (B) Downregulation of the lncRNA TCONS_00230836 in AML12 cells under stearic acid treatment. *n* = 5 mice per group; *n* = 4 independent cell cultures. Ctrl, control group; SA, stearic acid; HSD, high-stearic-acid diet. ^*^*P* < 0.05, ^**^*P* < 0.01 versus the Ctrl group.
