# Supplementary material for: TCONS_00230836 silencing restores stearic acid-induced β cell dysfunction through alleviating endoplasmic reticulum stress rather than apoptosis
Source: Genes Nutr. 2021 May 22;16:8. doi: 10.1186/s12263-021-00685-5 (PMC8140511; doi:10.1186/s12263-021-00685-5)
Supplement: Supplementary file 5 — Additional file 5. The intracellular insulin content in β-TC6 cells after transfection of the TCONS_00230836 Smart Silencer in the absence or presence of stearic acid. n = 3 cell cultures per group. Ctrl, control group; SA, stearic acid, si-lnc836, Smart Silencer for TCONS_00230836. [file 12263_2021_685_MOESM5_ESM.docx]

**Additional file 5**


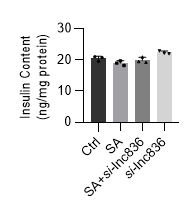


The intracellular insulin content in β-TC6 cells after transfection of the TCONS_00230836 Smart Silencer in the absence or presence of stearic acid. *n* = 3 cell cultures per group. Ctrl, control group; SA, stearic acid, si-lnc836, Smart Silencer for TCONS_00230836.
