# Supplementary material for: TCONS_00230836 silencing restores stearic acid-induced β cell dysfunction through alleviating endoplasmic reticulum stress rather than apoptosis
Source: Genes Nutr. 2021 May 22;16:8. doi: 10.1186/s12263-021-00685-5 (PMC8140511; doi:10.1186/s12263-021-00685-5)
Supplement: Supplementary file 6 — Additional file 6. The log2 fold change of up- and down-regulated differentially expressed mRNAs related to lncRNA TCONS_00230836 (PCC ≥0.950, or PCC ≤ -0.950, P < 0.05) in stearic acid-induced β-TC6 cells by high-throughput RNA-sequencing. [file 12263_2021_685_MOESM6_ESM.docx]

**Additional file 5**

The log_2_ fold change of up- and down-regulated differentially expressed mRNAs related to lncRNA TCONS_00230836 (PCC ≥0.950, or PCC ≤ -0.950, *P* < 0.05) in stearic acid-induced β-TC6 cells by high-throughput RNA-sequencing.

| Ensemble gene ID | Gene name | Log_2_ fold change |
| --- | --- | --- |
| ENSMUST00000074898 | *Hp*(Haptoglobin) | 6.940 |
| ENSMUST00000086399 | *Icam1*(intercellular adhesion molecule 1) | 6.190 |
| ENSMUST00000006956 | *SAA3*(Serum amyloid A3) | 6.127 |
| ENSMUST00000050785 | *Lcn2*(lipocalin 2) | 5.881 |
| ENSMUST00000159720 | *Alpk1*(alpha-kinase 1) | 4.294 |
| ENSMUST00000131456 | *Serping1*(serine (or cysteine) peptidase inhibitor, member 1) | 4.205 |
| ENSMUST00000023994 | *Serping1* | 4.044 |
| ENSMUST00000020190 | *Vnn3*(vanin 3) | 3.151 |
| ENSMUST00000027748 | *Rgs16*(regulator of G-protein signaling 16) | 3.062 |
| ENSMUST00000181286 | *Gm16685* | 2.886 |
| ENSMUST00000022531 | *Lats2*(large tumor suppressor 2) | 2.445 |
| ENSMUST00000036188 | *Zc3h12a*(zinc finger CCCH type containing 12A) | 2.419 |
| ENSMUST00000124100 | *Prn*(prion protein gene) | -5.858 |
| ENSMUST00000092822 | *Bcas3*(breast carcinoma amplified sequence 3) | -5.560 |
| ENSMUST00000021620 | *Otub2*(OTU domain, ubiquitin aldehyde binding 2) | -5.161 |
| ENSMUST00000073388 | *Afmid*(Arylformamidase) | -4.336 |
| ENSMUST00000149884 | *Snapin*(SNAP-associated protein) | -4.299 |
| ENSMUST00000185596 | *Srgap2*(SLIT-ROBO Rho GTPase activating protein 2) | -3.567 |
| ENSMUST00000142821 | *2010107G23Rik*(RIKEN cDNA 2010107G23 gene) | -3.541 |
| ENSMUST00000106372 | *Sult1a1*(sulfotransferase family 1A, phenol-preferring, member 1) | -2.866 |
| ENSMUST00000159089 | *Ipo9*(Importin 9) | -2.503 |
| ENSMUST00000135138 | *Gm15350* | -2.168 |
| ENSMUST00000128218 | *Gm16272* | -2.049 |
| ENSMUST00000111398 | *Ncor2*(nuclear receptor co-repressor 2) | -1.977 |
| ENSMUST00000135176 | *Gm14296* | -1.939 |
| ENSMUST00000142159 | *Ush2a*(Usher syndrome 2a) | -1.827 |
| ENSMUST00000133573 | *Rft1*(RFT1 homolog) | -1.111 |
